# Limited genetic variability and spatial population structure in grasshoppers between natural and metal-contaminated areas in Egypt

**DOI:** 10.1093/jisesa/ieae026

**Published:** 2024-03-19

**Authors:** Mustafa Soliman, Abdulrhman Almadiy, Rasha Al-Akeel, Thomas Hesselberg, Amr Mohamed

**Affiliations:** Department of Entomology, Faculty of Science, Cairo University, Giza 12613, Egypt; Department of Biology, College of Science and Arts, Najran University, Najran 1988, Saudi Arabia; Department of Zoology, Faculty of Science, King Saud University, Riyadh 11451, Saudi Arabia; Department of Biology, University of Oxford, Oxford, UK; Department for Continuing Education, University of Oxford, Oxford, UK; King Saud University Museum of Arthropods, Plant Protection Department, College of Food and Agricultural Sciences, King Saud University, Riyadh 11451, Saudi Arabia; Division of Invertebrate Zoology, American Museum of Natural History, 200 Central Park West, New York, NY 10024, USA

**Keywords:** RAPD-PCR, genetic variability, *Aiolopus thalassinus*, heavy metal pollution, geographical distances

## Abstract

Pollutants in an environment can have long-term implications for the species living there, resulting in local adaptations with implications for their genetic structure. Heavy metal pollutants infiltrate soils and groundwater, bioaccumulate in food webs, and negatively impact biota. In this study, we investigated the degree to which the genetic structure and variability of the slender green-winged grasshopper (*Aiolopus thalassinus* (Fabricius) (Orthoptera: Acrididae)) were impacted by heavy metal pollution and distance. We used the random amplified polymorphic DNA-polymerase chain reaction (RAPD-PCR) method to examine the genetic variability of populations in 3 heavy metal-polluted and 3 unpolluted locations across varying geographical distances in Egypt. The heavy metal concentrations of cadmium, copper, lead, and zinc were measured from the grasshopper tissue and soils. Sixty-nine unique and polymorphic bands were produced by 4 primers. Cluster and principal component analyses separated the populations inside and outside Cairo into 2 main branches, which were further divided into smaller branches corresponding to their geographical regions. We found no differences in the Shannon genetic diversity index between populations or with increasing heavy metal concentrations in either the soil or the grasshopper tissue. Our results showed a greater genetic variation among populations than between populations within the same location, indicating populations within locations were less differentiated than those between locations. The moderate correlation between genetic similarity and spatial distance suggests geographical isolation influenced grasshopper population differentiation. Based on the RAPD analysis, environmental pollutants and geographical distances impact the *A. thalassinus* population structure, potentially restricting gene flow between sites even at small spatial scales.

## Graphical Abstract

**Graphical Abstract:**
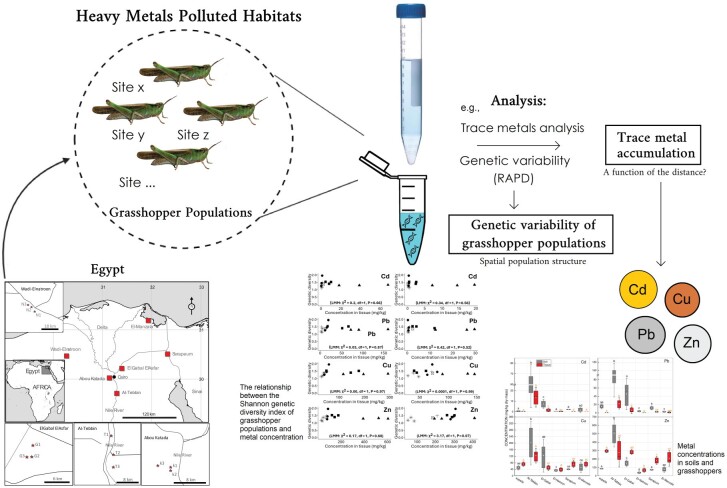

## Abstract (ar)

الملخص باللغة العربية

يمكن أن يكون لوجود الملوثات في بيئة ما آثار طويلة المدى على الأنواع التي تعيش فيها، ما يؤدي إلى تكيفات محلية لتلك الأنواع مع تأثيرات على بنيتها الجينية. تُلوث المعادن الثقيلة التربة والمياه الجوفية، وتتراكم أحيائياً في الشبكات الغذائية، وتؤثر سلباً على الكائنات الحية. في هذه الدراسة، تمت مراجعة تأثير الملوثات البيئية على التنوع الوراثي والتركيب الجيني للجندب النحيف ذو الأجنحة الخضراء ( *أيولوبوس ثالاسينوس * (فابريسيوس) (مستقيمات الأجنحة: الجنادب))، والذي يُعد مؤشرًا على صحة البيئة. تمت دراسة التركيب الوراثي والتنوع في آهلات هذا النوع في مواقع ملوثة بالمعادن الثقيلة ومواقع غير ملوثة عبر مسافات جغرافية في نفس الدولة. تم استخدام التحليل البلمري المتسلسل للحمض النووي الصبغي المتعدد الأشكال العشوائي لدراسة التباين الوراثي بين الآهلات في هذه المواقع المختلفة. تم قياس تركيزات المعادن الثقيلة من الكادميوم والنحاس والرصاص والزنك من أنسجة الجندب. تضمنت التحاليل الإحصائية للبيانات المتحصل عليها التحليل العنقودي والتحليل عبر المركبات الرئيسية ونموذج التأثير المختلط الخطي. أظهرت النتائج أن هناك تباينًا جينيًا أكبر بين مجموعات الآهلات من المناطق الجغرافية المختلفة مقارنة بتلك الموجودة داخل نفس المنطقة، مما يشير إلى وجود تمايز محلي بسبب التلوث البيئي. تشير النتائج أيضًا إلى أن الانعزال الجغرافي قد أثر على تمايز آهلات هذا النوع. وبناءً على التحليل الوراثي، أظهرت النتائج أن الملوثات البيئية والمسافات الجغرافية تؤثر على تركيبة آهلات هذا النوع، مما يشير إلى أن تدفق الجينات بين المواقع قد يكون مقيدًا حتى على المستويات المكانية الصغيرة. وتوصلت الدراسة إلى أن الملوثات البيئية تؤثر على التركيب الجيني والتنوع الوراثي للنوع المدروس، وقد يكون لها تأثير طويل المدى على التكيفات المحلية والتغير في البنية الجينية. ومن ثم، تقدم هذه الدراسة نظرة مُحْكَمة للاستجابات الجينية للجندب تحت الدراسة للتلوث البيئي من خلال منهجية التحليل الوراثي القوية.

## Introduction

Wide-ranging effects of environmental pollution include contaminating the atmosphere, soil, and water, as well as exposing all living things to a wide range of potential stressors, including heavy metal and metalloid contaminants (Wasi et al. 2013). The impacts of heavy metal contamination on different environmental settings and living systems are detailed elsewhere (Wu et al. 2016). Heavy metal contaminants are highly reactive and frequently toxic in low concentrations and have been shown to be able to travel long distances (Briffa et al. 2020). This implies that habitats and species from less anthropogenically disturbed regions—that is, places with less agriculture, developed industries, or urbanization—are also susceptible to pollution. Overall, the significant changes that humans have brought about to ecosystems include the fast loss of biodiversity, the geographic relocation of species, and biotic homogenization (Sigmund et al. 2023). These factors, taken together, may cause the sixth mass extinction to occur on Earth (Cowie et al. 2022).

A straightforward and efficient way to track down the sources of heavy metal pollution involves first conducting a sampling analysis in the areas (geolocations) where pollution sources are present. Geographic information system techniques are used to map specific areas or settings for contamination (Hou et al. 2017). These include using the global positioning system to obtain longitude and latitude coordinates and may involve advanced remote sensing techniques. The geostatistical method can then effectively and usefully describe the spatial variation of the source data by interpolating a number of data points. Spatial variation function models (exponential function models, Gauss, spherical, etc.) and spatial interpolation techniques (such as kriging, ordinary, and simple) are among the models the geostatistical method comprises (Hou et al. 2017, Kumar et al. 2019). The impact of pollution sources (source distance and buffering zones, land use, urban industry layouts, transportation roads, waste irrigation, mining areas, landfills, etc.) on soil heavy metal pollution is extensively studied through this type of research, which has shown enormous progress (Weissmannová and Pavlovský 2017, Wang et al. 2021). For example, monitoring studies on soil heavy metal contamination indicated that the distance between the sampling sites and the sources of pollution is a crucial parameter (Soliman et al. 2022). The amount of heavy metal pollution will typically decrease exponentially with distance from the source. Chemometric methods like correlation and principal component analysis (PCA) methods are used to identify pollution sources (Inobeme et al. 2022). Various studies have linked genetic and geographic distances effectively using the geolocation approach (Lagisz et al. 2010, Vidal et al. 2019, Li et al. 2023). But focusing solely on geolocation would only highlight patterns of isolation based on distance; adding pollution as a covariate enables researchers to examine the additional ways that anthropogenic environmental change affects gene flow and population connectivity (Pedrosa et al. 2017, Gouin et al. 2019).

Genetic changes in natural populations represent subtle outcomes of anthropogenic toxicants in the environment, with substantial long-term implications (Li et al. 2019). Various forms of pollution can significantly alter the genetic composition of populations and influence genetic variation through 4 primary mechanisms: (i) increased mutation rates; (ii) directional selection favoring tolerant genotypes; (iii) genetic bottlenecks; and (iv) altered migration patterns (Mussali-Galante et al. 2014). Numerous studies have examined contaminant exposure effects on the genetics of both terrestrial (Grzywacz et al. 2012, Giska et al. 2015, Renault et al. 2023) and aquatic (Gouin et al. 2019, 2023, Švara et al. 2022) populations. In addition to toxicants, natural phenomena like mutations, selection, stochastic processes, and migration can modify the genetic variability of populations (Mussali-Galante et al. 2014). Notably, gene flow is augmented through individual movement between populations, hybridization, and nucleotide substitutions, contributing to the complex dynamics shaping genetic variability (Ringbauer et al. 2018).

Comparing genotypes of the same species from polluted and unpolluted sites can reveal altered gene frequencies with physiological or ecological implications (Grzywacz et al. 2012). Advances in techniques directly revealing genetic variation at the DNA level have enabled exploring relationships between genetics and ecotoxicology for risk assessment (Williams et al. 1990, Babu et al. 2013, da Silva et al. 2023). The discovery that PCR with a single arbitrary primer can amplify varyingly sized DNA fragments in any genome facilitated the development of genetic markers (Williams et al. 1990, Babu et al. 2013). This technique has been widely used to identify polymorphisms in various organisms, including grasshoppers (Chapco et al. 1992, Silveira et al. 1998, Zhang and Kang 2005, Sesarini and Remis 2008, Miño et al. 2010, Jamal et al. 2020).

The popularity of random amplified polymorphic DNA (RAPD) stems from its ability to generate numerous polymorphic loci from minimal DNA without prior template knowledge (Babu et al. 2013). RAPD markers effectively distinguish geographically and genetically isolated populations within species, elucidating populations arising through drift or selection under varying conditions (Vaughn and Antolin 1998, González et al. 2007, Kil 2015, Mohammadi Sharif et al. 2022). In Egypt, *Aiolopus thalassinus* is a predominant grasshopper species with wide Palearctic, Afrotropical, Oriental, and Australian distribution (Hollis 1968, GBIF Secretariat 2023). Both nymphs and adults occur year-round, feeding on plants and providing food for diverse predators (Schmidt 1986, Mückstein and Vlk 2015).

This study investigates heavy metal accumulation in *A. thalassinus* across an industrial pollution gradient. A previous study has shown that *A. thalassinus* serves as an ecological bioindicator for Cd, Pb, Cu, and Zn pollution (Yousef et al. 2017). Stable populations at contaminated sites suggest a remarkable heavy metal tolerance through efficient detoxification and/or adaptation. However, the extent of genetic variation remains unknown. Therefore, the present study aimed to analyze genetic diversity among *A. thalassinus* populations from polluted and unpolluted locations across geographical distances. Through this first exploration of the genetics of *A. thalassinus*, we further aimed to determine whether the genetic similarity between populations correlates with their proximity and to identify any associations between genetic diversity and environmental pollution levels.

## Materials and Methods

### Sampling Sites

**Fig. 1.**
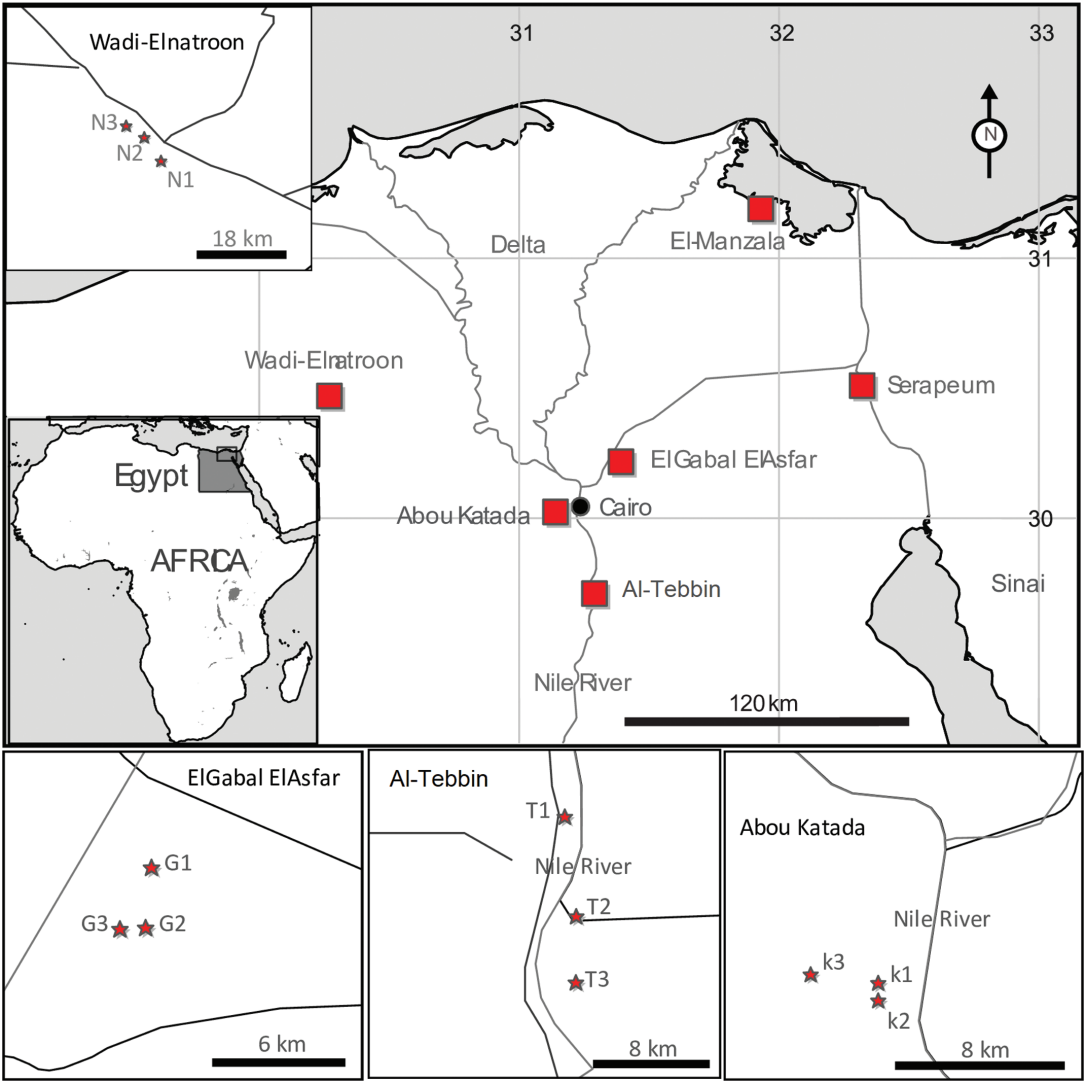
A map of Egypt displays the locations where populations were collected.

Specimens of the slender green-winged grasshopper (*A. thalassinus*) were sampled from 6 metapopulations with varying heavy metal pollution. Three populations were situated in areas associated with higher pollution in Greater Cairo: Al-Tebbin (31.293547°N, 29.762689°E), El-Gabal El-Asfar (31.374058°N, 30.217489°E), and Abou Katada (31.195571°N, 30.031577°E). Three additional populations from other governorates—Wadi El-Natroon (30.303553°N, 30.424499°E) in Al-Beheira, Serapium (32.23665°N, 30.48085°E) in Ismailiya, and El-Manzala (31.792649°N, 31.387418°E) in Dakahlieh—were considered relatively unpolluted (Fig. 1). Greater Cairo encompasses 928 km^2^ near the Nile River within Cairo, Giza, and Qaliubiah governorates (El Araby 2002).

### Collection and Preparation of Samples

Using insect nets, 200 adult *A*. *thalassinus* specimens of both sexes were collected from agroecosystems during the summer season. Specimens were transported to the laboratory alive, killed by freezing, and then stored at −20 °C for 6 months before analysis. Concurrently, upper soil samples (0–15 cm) were randomly obtained from each site, thoroughly mixed, and stored in plastic bags. Insect and soil samples were dried at 70 °C to a constant mass, ground to a fine powder, and stored at room temperature. Before grounding insect samples, one leg was dissected from the first thoracic segment of each insect specimen for DNA extraction. We used sterile dissection tools, cleaned with a 10% sodium hypochlorite solution, followed by 70% ethanol between each sample to prevent cross-contamination.

### Trace Metals Analysis

*Aiolopus thalassinus* samples were digested according to Soliman et al. (2022). After predigestion with 10 ml of 65% HNO_3_ for 24 h at room temperature, 0.5 g of insect samples were further digested and evaporated at 90 °C to near dryness. Then, 2 ml of 30% H_2_O_2_ was added and reheated to near dryness. Flask walls were washed with 10 ml of ultrapure water, filtered through Whatman #41 filter paper into a 25-ml volumetric flask, diluted, and stored at 4 °C until analysis. Soil samples underwent similar digestion but stood for 24 h with a 12 ml of 37% HCl:65% HNO_3_ (3:1) mixture. Then, 2.5 ml of 37% HCl and 2.5 ml of 30% H_2_O_2_ were added to complete digestion before dilution to 50 ml. Metal (Cd, Pb, Cu, and Zn) concentrations were determined by inductively coupled plasma (ICP-AES). All glassware was acid-washed prior to use. Chemicals were analytical grade (Merck, Darmstadt, Germany).

### DNA Extraction and RAPD Amplification

The recommendations of Black and DuTeau (1997) for efficient RAPD-PCR analysis in genetic studies involving insect populations were closely followed. To prevent contamination, DNA extraction and PCR setup were performed in separate, dedicated rooms using dedicated pipettes. Filter tips were used for all pipetting. Genomic DNA was pooled and extracted from 9 to 20 legs/population to avoid cross-contamination with foreign DNA using the QIAamp DNA mini kit (Qiagen, Hilden, Germany) per the manufacturer. Twenty RAPD primers (Operon Technologies, Alameda, California, USA) were tested (Supplementary Material 1). Amplification was performed using GoTaq Flexi DNA polymerase (Promega, Madison, Wisconsin, USA). Reaction mixtures contained 50 ng DNA, 5× Green Buffer, 0.75 mM MgCl_2_, 1.25 U polymerase, 30 pmol primer, and 0.5 mM dNTPs in a 25-μl total volume. Thermocycling comprised initial denaturation at 94 °C for 4 min, then 40 cycles of denaturation at 94 °C for 45 s, annealing at 36 °C for 1 min, and extension at 72 °C for 2 min, followed by final extension at 72 °C for 7 min. Amplified products were separated by electrophoresis on 1.5% agarose gels alongside a 100-bp ladder (Promega, Madison, Wisconsin, USA), stained with GelRed (Biotium, Fremont, California, USA), and visualized under UV. Images were digitally captured (BioRad Gel Doc; Bio-Rad Laboratories, Inc., California, USA).

### Data Analysis

Each DNA fragment generated using RAPD primers was regarded as a binary unit character. A score of “1” was assigned for the presence of a fragment, while “0” denoted its absence. To assess the informativeness of these primers, the discriminatory power percentage was computed as the ratio of the number of unique bands produced by each primer to the total number of unique bands across all primers, following the methodology outlined by Grundmann et al. (1995). Furthermore, the Shannon genetic diversity index was calculated for the amplified nucleotide fragments within each population, employing 4 distinct primers, namely OP N-02, OP H-08, OP J-11, and OP D-08. The computational analysis was performed using BioNumerics software version 7.5 (Applied Maths NV, Belgium).

Shannon diversity index *Hs* was calculated as:


$$\begin{array}{l} Hs=\underset{i=1}{\overset{n}{\sum }}-\frac{hi}{H}ln\frac{hi}{H} \end{array}$$

Here, “*n*” represents the total number of bands within the population, “*hi*” signifies the intensity of each individual band “*i*,” and “*H*” denotes the cumulative intensity of all bands in the population. Since its implementation in population genetics research in the 1970s, the Shannon index has been extensively utilized and validated as a metric of genetic diversity across populations (Konopiński 2020). Jaccard’s coefficient of similarity was also calculated using BioNumerics version 7.5 based on the presence and absence of amplified DNA fragments. The formula for Jaccard’s coefficient is as follows:


$$\begin{array}{l} J=\frac{a}{a+b+c} \end{array}$$

where “*a*” is the number of bands present in both populations, “*b*” is the number of bands present only in population 1, and “*c*” is the number of bands present only in population 2. Subsequently, a dendrogram was constructed to visually represent the relationships among all populations and subpopulations based on scorable bands from primer OPN-02. This dendrogram was generated using the unweighted pair group method of arithmetic mean (UPGMA). For a more comprehensive understanding of the genetic variation at 2 hierarchical levels, namely the population and subpopulation, an analysis of molecular variance (AMOVA) was carried out using the Arlequin software version 3.5 (Excoffier and Lischer 2010). Principal component analysis (PCA) was conducted on all populations and subpopulations to assess their geographical relationships. The Shannon genetic diversity index was compared between populations of polluted and control sites with a linear mixed effect model (LMM) with the site as a random variable using the *lmer()* function in R (R version 4.2.2; 2022). *P*-values were estimated with the Type II Wald chi-square tests. Similar LMMs were developed to test the relationship between heavy metal pollution levels (in both soil and grasshopper tissue) and Shannon genetic diversity index values. The Kruskal-Wallis *H*-test was employed to assess significant variations in metal concentrations (in both soil and tissue) among the 6 locations. This analysis was then followed by conducting pairwise comparisons using the Mann–Whitney *U*-test. The nonparametric Spearman’s rank correlation test was used between geographical distances and Jaccard’s similarity coefficients for the populations. PCA, Kruskal–Wallis *H*-test, Mann-Whitney *U*-test, and correlation analyses were executed using IBM SPSS Statistics Version 27 software (IBM Corp., Armonk, NY, USA).

## Results and Discussion

### Trace Metal Accumulation

**Fig. 2.**
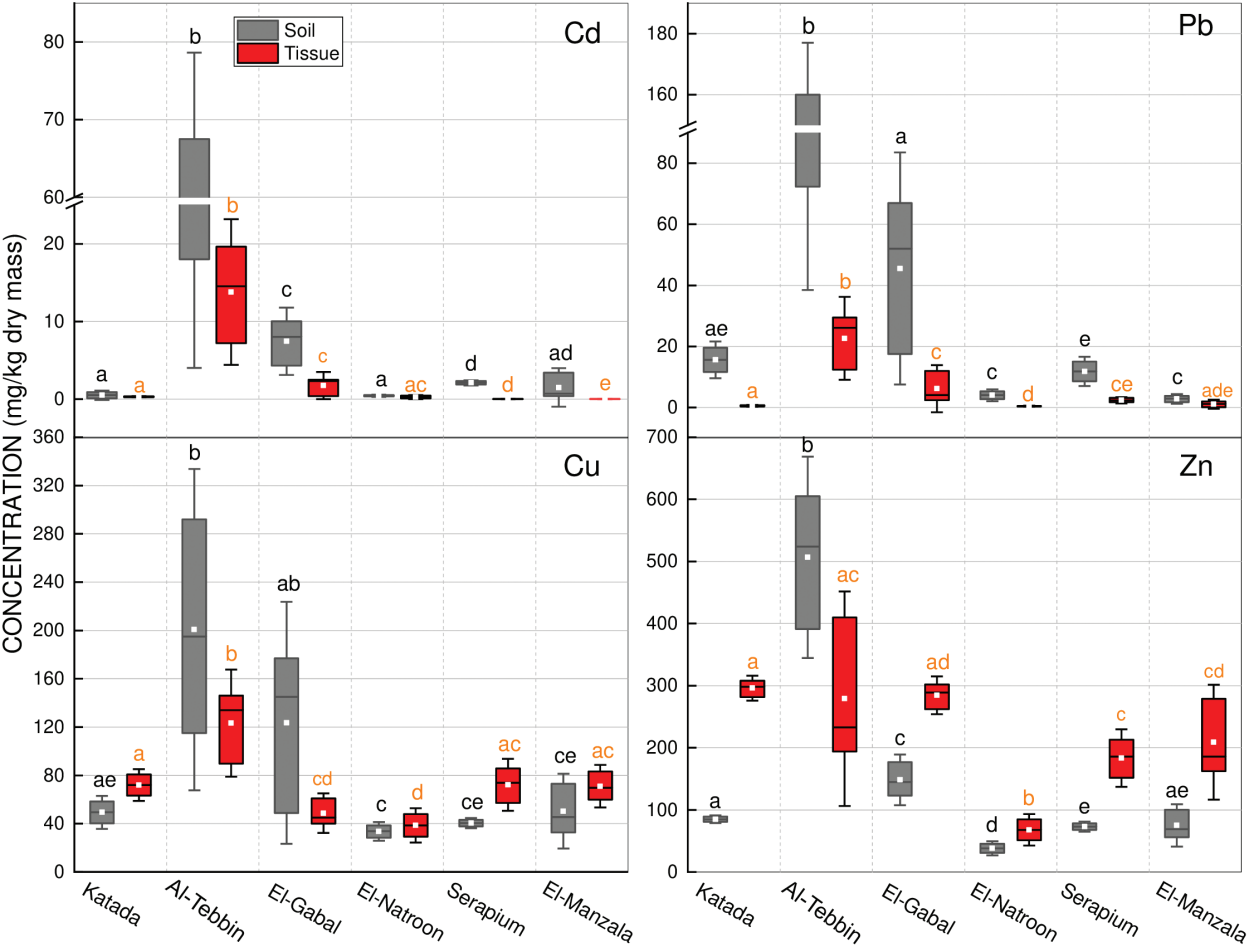
Metal concentrations in soils and grasshoppers. Data are presented as mean ± SD in mg/kg dry weight. Boxes followed by different black letters denote significant differences in metal concentrations in soils between the 6 locations (Mann–Whitney *U*-test, *P* ≤ 0.05). Boxes followed by different orange letters denote significant differences in metal concentrations in grasshopper tissues between the different sites (Mann–Whitney *U*-test, *P* ≤ 0.05).

Metal concentrations were generally lower in grasshopper tissues than in corresponding soil samples, except for Cu and Zn at most sites (Fig. 2). However, statistically significant differences in metal concentrations occurred between the 6 locations for both soil and tissue samples (*H* = 12.485–15.690, *P* < 0.05 for soil; *H* = 12.789–15.418, *P* < 0.05 for tissues). Focusing on specific geographical areas, Al-Tebbin and El-Gabal Al-Asfar in Greater Cairo exhibited higher heavy metal levels in soil and grasshopper tissues compared to other sites. This was especially evident for Al-Tebbin. Alternatively, locations outside Cairo (Wadi El-Natroon, Serapium, and El-Manzala) showed relatively lower concentrations, with Wadi El-Natroon having the lowest levels. Previous research thoroughly documented heavy metal exposure in these polluted areas. Al-Tebbin lies in an industrial zone south of Cairo (Fig. 1) with metallurgical, chemical, and cement production (Gomaa et al. 2020). Since 1923, El-Gabal Al-Asfar has received irrigation from wastewater effluent (Gemail 2012). Heavy metal accumulation in these soils poses major environmental threats.

### RAPD-PCR Analysis of the *A*. *thalassinus* Populations

Twenty carefully selected primers were used to evaluate polymorphism among 14 *A. thalassinus* populations. Only 4 primers produced clear amplification, while 16 failed to amplify. Consequently, all populations were analyzed with OP N-02, OP H-08, OP J-11, and OP D-08 (Supplementary Material 1). Each primer underwent duplicate amplification, considering only bright, reproducible bands. These 4 primers generated 69 scorable polymorphic bands within 190–2,111 bp, averaging 17 bands per primer and effectively discriminating populations. Primer OP N-02 produced the most bands (23), while OP D-08 yielded the fewest (9). Primer OP J-11 generated the largest band (2111 bp), and OP H-08 produced the smallest (189.71 bp). All primers consistently detected polymorphisms (100% success rate). In terms of discriminatory power, percentages were 33.3% for OP N-02, 28.9% for OP H-08, 24.6% for OP J-11, and 13.0% for OP D-08. With 23 polymorphic bands, OP N-02 was the most informative primer and was selected for comparing *A. thalassinus* populations, which showed distinct band patterns.

### Genetic Diversity and Differentiation of *A*. *thalassinus* Populations

**Fig. 3.**
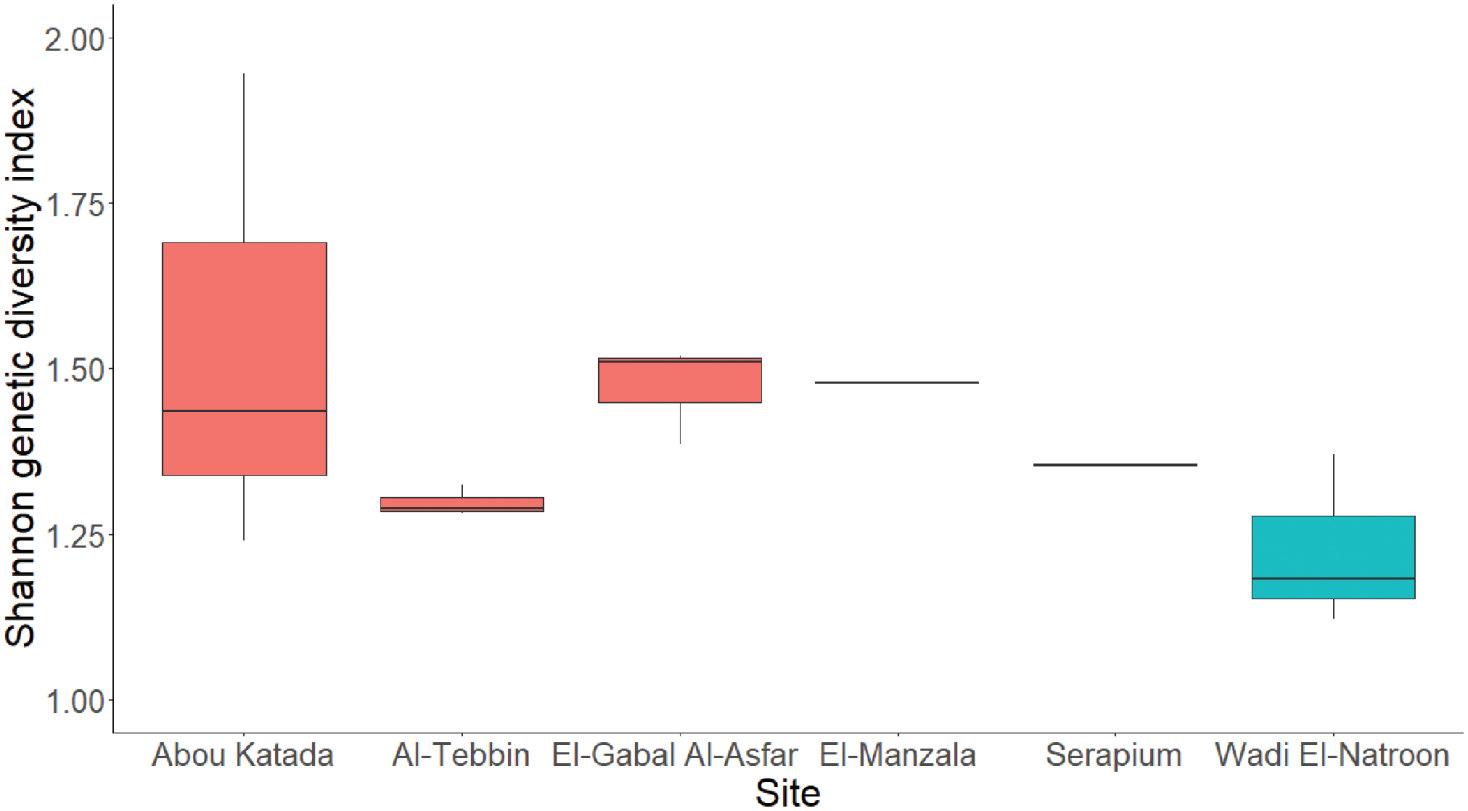
The genetic diversity index of *Aiolopus thalassinus* populations averaged per site (*N* = 3 populations per site, except for El-Manzala and Serapium, which had *N* = 1 population each). The first 3 sites (colored red) are the polluted sites, while the last 3 (colored blue) are the nonpolluted sites. The Shannon genetic diversity index was calculated for the amplified nucleotide fragments of each population in the 4 primers (OP N-02, OP H-08, OP J-11, and OP D-08).

**Fig. 4.**
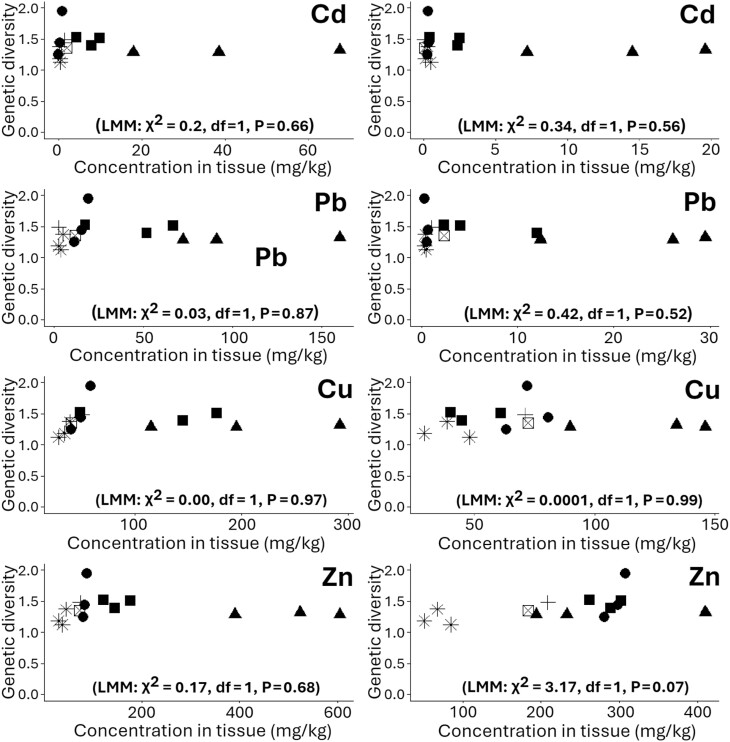
The relationship between the Shannon genetic diversity index of *Aiolopus thalassinus* populations and metal concentrations (measured in mg metal per kg dry mass). The figures on the left hand show the metal concentration in soil, and the figures on the right hand show the metal concentration in grasshopper tissue (*N* = 14 for each). The symbols indicate the different sites (stars—Wadi El-Natroon, crosses—El-Manzala, squares—El-Gabal Al-Asfar, ticked squares—Serapium, circles—Abou Katada, triangles—Al-Tebbin). The Shannon genetic diversity index was calculated for the amplified nucleotide fragments of each population in the 4 primers (OP N-02, OP H-08, OP J-11, and OP D-08).

The potential impacts of toxicants on genetic diversity within natural populations encompass a wide spectrum (Mussali-Galante et al. 2014, Dornbos and LaPres, 2018, Li et al. 2023). It has been documented that populations exposed to heavy metal pollution can exhibit varying responses, leading to either an increase or decrease in their genetic variation. An illustrative case can be found in the long-horned groundhopper, *Tetrix tenuicornis* (Sahlberg) (Orthoptera: Tetrigidae), where individuals collected from metal-polluted regions in Boleslaw, Poland, displayed reduced genetic variability compared to populations from unpolluted areas (Grzywacz et al. 2012). Conversely, in a study by Giska et al. (2015), the highest genetic diversity was observed in the rove beetle, *Staphylinus erythropterus* Linnaeus (Coleoptera: Staphylinidae), populations inhabiting a heavily metal-polluted site in southern Poland. However, our study did not find any differences in genetic diversity between heavy metal-polluted sites and control sites (LMM: χ^2^ = 1.33, df = 1, *P* = 0.25), except that the control site Wadi El-Natroon appeared to have lower genetic diversity than other sites (Fig. 3). Similarly, none of the metals analyzed in both soil and *A. thalassinus* showed a significant correlation with population genetic diversity (all linear mixed effect models had *P* > 0.05) (Fig. 4).

Based on RAPD markers, a UPGMA dendrogram was constructed using Jaccard’s similarity coefficient to visually depict the interrelationships among populations of *A. thalassinus* (Supplementary Material 2). Six populations formed 5 major branches, closely corresponding to their respective geographical distributional regions: Al-Tebbin, Abou Katada, El-Gabal, Wadi El-Natroon and Serapium, and El-Manzala. Subsequent exploration of gene flow among *A. thalassinus* populations was undertaken by analyzing population genetic structures. An AMOVA analysis revealed that genetic variation among populations (67.6%) was greater than the variation between subpopulations (32.4%), indicating less genetic differentiation between populations within regions compared to those between regions (Table 1).

**Table 1.** AMOVA (molecular analysis of variance) output for 4 geographic populations of *Aiolopus thalassinus* (K, T, G, and N) relying on RAPD markers

Source of variation	df	SSD	Variance component	% total variance	*P*-value
Among populations	3	26.28	2.73	67.60	<0.0001
Among subpopulations	7	9.16	1.30	32.40	<0.0001
Total	10	35.45	4.04		

Statistics included degrees of freedom (df), sum of squared differences (SSD), variance component estimates, percentages of total variance (% total variance) contributed by the variance component, and the probability (*P*) of obtaining a more extreme component estimate by chance. The *P*-value is estimated from 1,023 sampling permutations.

Migration likely played an important role in gene flow and genetic diversity across geographical populations. Thus, the relatively high genetic variance among populations in our study could be explained by the absence or short-distance migration of *A. thalassinus* (Heifetz and Applebaum 1995). Our findings were similar to those for the redlegged grasshopper *Melanoplus femurrubrum* (De Geer) (Orthoptera: Acrididae) (Chapco et al. 1992), where genetic variability between populations was slightly higher than within populations. However, this contrasts with the migratory locust *Locusta migratoria* (Linnaeus) (Orthoptera: Acrididae) (Zhang and Kang 2005), which showed no difference in genetic variability between populations, likely due to its migratory nature.

Geographic isolation is a key factor contributing to population differentiation, as greater geographical distances between populations lead to decreased gene flow and increased differentiation (Liu et al. 2019). However, in the present study, clustering analysis divided the 6 regional populations into 2 primary clusters: The first cluster comprised populations from Greater Cairo (Al-Tebbin, El-Gabal, and Abou Katada), while the second cluster consisted of populations located outside of Cairo (Wadi El-Natroon, Serapium, and El-Manzala) (Supplementary Material 2). Despite the limited geographical area encompassed, all of the Cairo populations exhibited differentiation from one another, indicating constrained gene flow between them. Furthermore, the populations of Wadi El-Natroon and Serapium, which are situated relatively far apart from each other yet in proximity to the Cairo populations (Fig. 1), clustered with the northern population of El-Manzala in the UPGMA dendrogram. This implies that local conditions such as pollution, in addition to geographical distances, exert a substantial influence on the *A. thalassinus* population structure and may profoundly impact gene flow between sites, even on a very small spatial scale. Moreover, inhabiting an anthropogenic environment, such as an urban landscape, can potentially impact the dispersal ability of organisms. Urban areas often present barriers to dispersal, such as buildings, roads, and other infrastructure, which can limit gene flow between populations (Johnson and Munshi-South 2017). In contrast, rural areas may provide a more conserved and interconnected environment, facilitating gene flow and reducing genetic differentiation between populations.

**Fig. 5.**
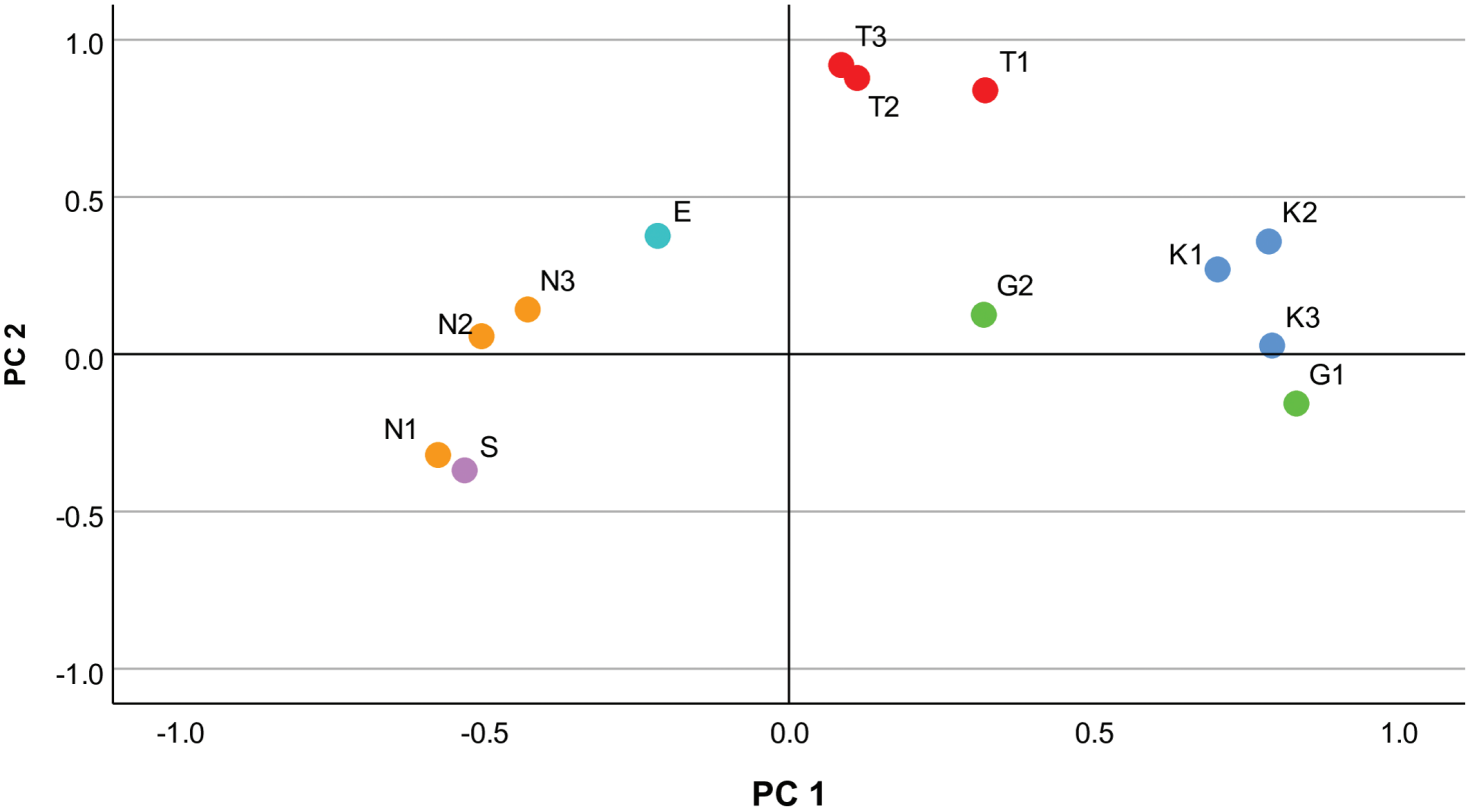
Principal components chart for 13 geographic populations and subpopulations of the *Aiolopus thalassinus*: Abou Katada (K1, K2, and K3), Al-Tebbin (T1, T2, and T3), El-Gabal Al-Asfar (G1 and G2), Wadi El-Natroon (N1, N2, and N3), Serapium (S), and El-Manzala (E).

Alterations in genetic variability can also result from adaptation to contaminated environments and prolonged exposure to genotoxic chemical agents (Mussali-Galante et al. 2014, Gouin et al. 2019). Mutations that impede primer binding or otherwise interfere with the amplification process can reveal this variation when using the RAPD technique (Butovskaia et al. 2009). By evaluating DNA polymorphism, researchers can identify population genetic responses to toxicant exposure (Grzywacz et al. 2012, Babu et al. 2013). Therefore, the RAPD analysis employed in this study has the potential to detect genetic changes in *A. thalassinus* related to adaptation and response to pollution and contamination in their habitats. As per the World Health Organization (2006), Cairo has gained recognition as one of the most severely air-polluted cities globally. Studies carried out by the National Environmental Board have revealed that the annual average of the air quality index in Cairo surpasses the maximum acceptable tolerance level by 3-fold (UNCT 2022). Considering this significant environmental challenge, it is plausible to speculate that a portion of the observed genetic variation in *A. thalassinus* populations can be attributed to the selective pressures exerted by the presence of environmental pollutants in Cairo. Principal component analysis (PCA) provided additional support for the population division observed in the cluster analysis (Fig. 5). The PCA results revealed notable distinctions between populations within and outside Cairo. Moreover, the population of Al-Tebbin, recognized as one of Egypt’s most significant industrial zones, exhibited discernible dissimilarity from the other 2 populations within Cairo.

**Fig. 6.**
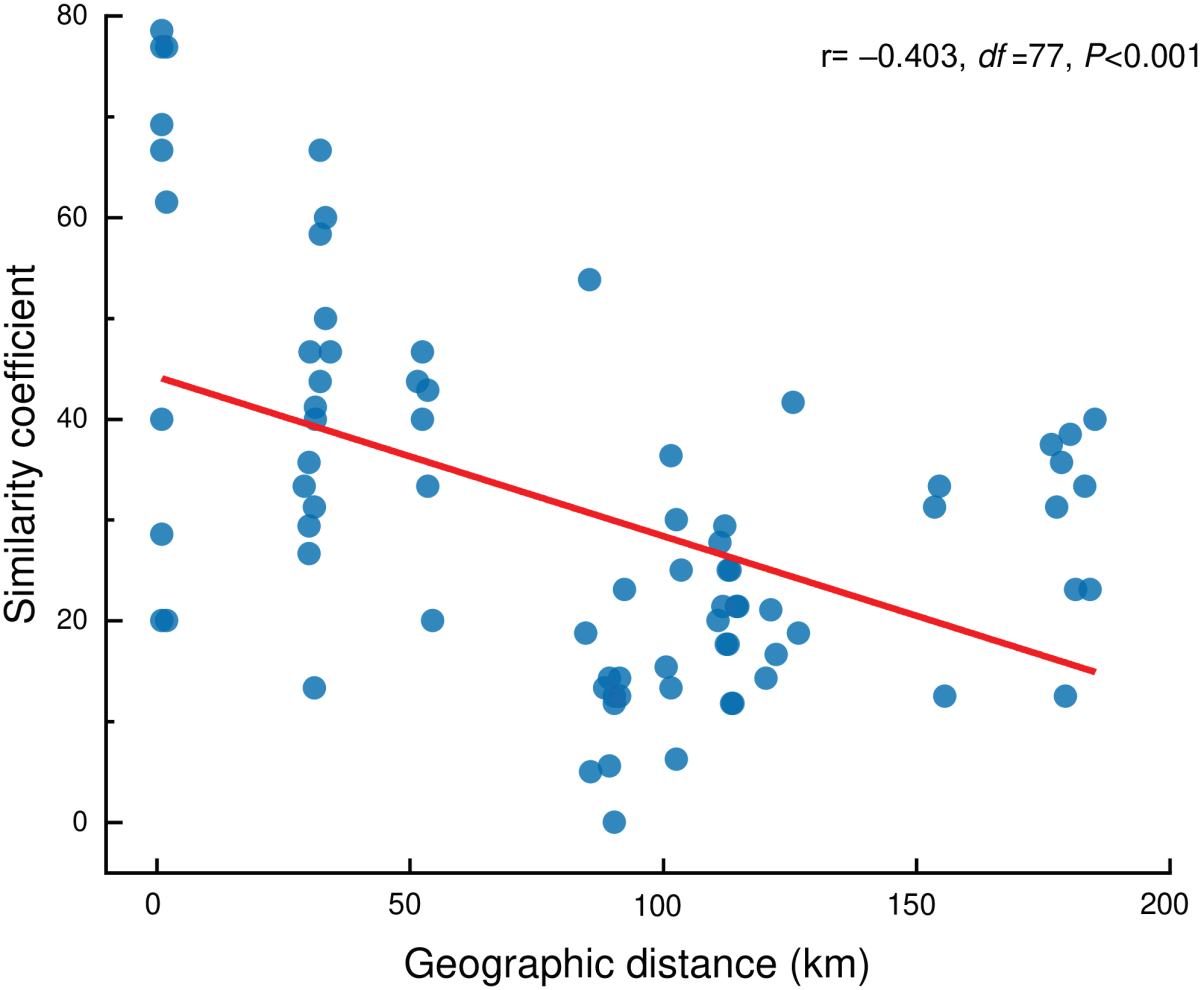
The correlation between Jaccard’s similarity coefficients of *Aiolopus thalassinus* populations based on scorable bands from primer OPN-02 and their corresponding geographic distances. The values of the Spearman’s rank-order correlation coefficient test (*r*), degree of freedom (df), and probability (*P*) are listed above.

Our RAPD analysis suggests the occurrence of gene flow among the 3 populations located outside the Greater Cairo area. In contrast, our observations indicate that gene flow within the populations of Greater Cairo may be relatively constrained. To investigate the potential geographic distribution of genetic diversity, we conducted Spearman’s rank correlation analysis between genetic similarity and geographical distances. This analysis aims to shed light on the influence of geographic separation on genetic diversity patterns within these populations. The results revealed a moderate level of correlation in the primer examined (*r* = –0.403, *P* < 0.001) (Fig. 6). These results suggest that population differentiation cannot be solely attributed to the degree of geographical isolation. While geographical distances may play a role, environmental pollutants likely contribute to the reduction of genetic variability in the *A. thalassinus* population.

The present findings provide supporting evidence for the assessment of interpopulation variability using RAPD analysis. Contrary to the hypotheses put forth by Ross et al. (2002), Zhiyi and Haowen (2004), and Maes et al. (2005), our data did not reveal a clear correlation between heavy metal pollution and genetic diversity. It is important to note that since multiple pollutants coexist in the environment, the individual impact of each metal on genetic diversity may not be easily discernible. Rather, the combined and synergistic effects of these pollutants may exert diverse levels of stress on populations. Therefore, in contrast to the conclusions drawn by these authors, it appears that both geographical distances and environmental pollutants, in general, influence the genetic diversity of *A. thalassinus* populations. Findings would aid in the genetic monitoring and ecological risk assessment of terrestrial ecosystems facing contamination. Further multiseasonal sampling and high-resolution genetic analysis of *A. thalassinus* across broader spatial scales would augment our understanding of adaptation, migration, and gene flow.

## Supplementary Material

ieae026_suppl_Supplementary_Materials_1

ieae026_suppl_Supplementary_Materials_2

## Data Availability

All data generated or analyzed during this study are included in this article and its supplementary information files.
