# Supplementary material for: Limited genetic variability and spatial population structure in grasshoppers between natural and metal-contaminated areas in Egypt
Source: J Insect Sci. 2024 Mar 19;24(2):12. doi: 10.1093/jisesa/ieae026 (PMC10949439; doi:10.1093/jisesa/ieae026)
Supplement: ieae026_suppl_Supplementary_Materials_1 [file ieae026_suppl_supplementary_materials_1.pdf]

Supplementary Material 1. Primers employed for PCR amplification of *Aiolopus thalassinus* populations

Primer	Sequence (5' to 3')	Number of bands	Size range of bands (bp)
OP B-09	TGGGGGACTC	0	0
OP C-10	TGTCTGGGTG	0	0
OP C-17	TTCCCCCCAG	0	0
OP D-08	GTGTGCCCCA	9	942-326.92
OP D-17	TTTCCCACGG	0	0
OP N-02	ACCAGGGGCA	23	1575-236.48
OP N-17	CATTGGGGAG	0	0
OP N-20	GGTGCTCCGT	0	0
OP E-03	CCAGATGCAC	0	0
OP E-13	CCCGATTCGG	0	0
OPAB-12	CCTGTACCGA	0	0
OP F-13	GGCTGCAGAA	0	0
OP H-08	GAAACACCCC	20	1459-189.71
OP J-11	ACTCCTGCGA	17	2111-430.63
OP J-15	TGTAGCAGGG	0	0
OP K-11	AATGCCCCAG	0	0
OP K-13	GGTTGTACCC	0	0
OP L-03	CCAGCAGCTT	0	0
OP L-08	AGCAGGTGGA	0	0
OP L-10	TGGGAGATGG	0	0
