# Supplementary material for: Limited genetic variability and spatial population structure in grasshoppers between natural and metal-contaminated areas in Egypt
Source: J Insect Sci. 2024 Mar 19;24(2):12. doi: 10.1093/jisesa/ieae026 (PMC10949439; doi:10.1093/jisesa/ieae026)
Supplement: ieae026_suppl_Supplementary_Materials_2 [file ieae026_suppl_supplementary_materials_2.pdf]

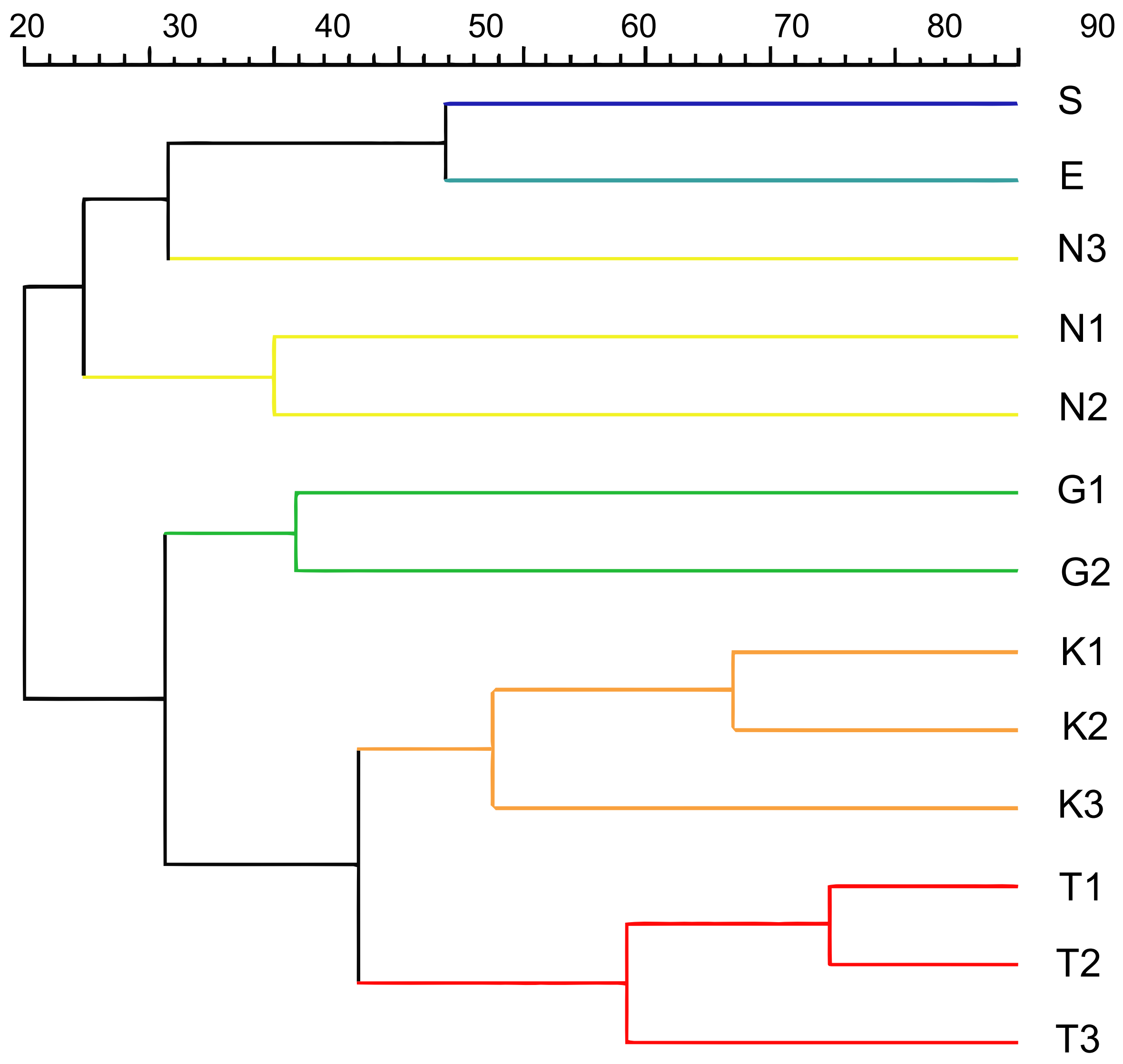

Supplementary Material 3. Dendrogram displaying the similarity among the populations and subpopulations of *Aiolopus thalassinus*, built with RAPD data (based on scorable bands from primer OPN-02) and attained by a Jaccard's coefficient using UPGMA. Abou Katada (K1, K2, and K3), Al-Tebbin (T1, T2, and T3), El-Gabal Al-Asfar (G1 and G2), Wadi El-Natroon (N1, N2, and N3), Serapium (S), and El-Manzala (E).
